# Simulated Self‐Assessment and Error Correction in Preclinical Prosthodontics: Insights From 3D‐Printed Models

**DOI:** 10.1111/eje.13131

**Published:** 2025-05-23

**Authors:** Constance Cuny, Abhishek Kumar, Alexandre Thuries, Geromine Fournier, Coralie Bataille, Cathy Nabet, Antoine Galibourg

**Affiliations:** ^1^ Faculty of Health, Department of Odontology Oral Rehabilitation Toulouse France; ^2^ Division of Oral Rehabilitation, Department of Dental Medicine Karolinska Institutet Huddinge Sweden; ^3^ Academic Center for Geriatric Dentistry Stockholm Sweden; ^4^ CNRS, Centre for Anthropobiology and Genomics of Toulouse, UMR 5288 Université Paul Sabatier Toulouse France; ^5^ INSERM UMR1295 Center for Epidemiology and Research in POPulation Health (CERPOP) Paris France

**Keywords:** 3D printing, error correction, fixed prosthodontics, pedagogy, self‐assessment

## Abstract

**Introduction:**

Developing self‐assessment and defect correction skills is essential for dental students to ensure high‐quality clinical practice. However, these skills are often insufficiently addressed in dental education. This study evaluates students' ability to assess and correct crown preparation defects through a structured exercise simulating self‐assessment using standardised 3D‐printed models with deliberate flaws. Additionally, it examines their improvement during the correction phase and their satisfaction with this innovative approach.

**Materials and Methods:**

Students evaluated crown preparation defects on standardised 3D‐printed models, guided by a rubric‐based evaluation table, and subsequently corrected these defects using rotary instruments. Their perceptions of the exercise were captured through a satisfaction questionnaire.

**Results:**

92% of students overestimated the presence or severity of defects during self‐assessment, with axial and occlusal reduction being the most challenging criteria. After the correction phase, students demonstrated significant improvement, particularly in axial reduction, although challenges persisted in occlusal reduction and finishing. Students expressed high satisfaction with the exercises, emphasising their value in enhancing learning and critical thinking.

**Conclusion:**

This study underscores the importance of structured self‐assessment and correction exercises in dental education. By bridging the gap between self‐assessment and self‐correction, particularly in crown preparations, 3D‐printed models foster the development of clinical skills and autonomy amongst students.

## 1 Introduction

Assessing students' readiness for clinical practice is a persistent concern in dental education, despite the structured foundation provided by preclinical curricula [1]. Developing self‐assessment skills early in their training helps students recognise their strengths and weaknesses, contributing to the refinement of their clinical performance [2, 3, 4, 5, 6]. Additionally, fostering these skills supports reflective practice, which promotes continuous learning and improvement in clinical settings.

In prosthodontics, preclinical training focuses on developing manual dexterity, a key clinical skill [7]. Traditionally, students' work is evaluated step‐by‐step by instructors using simulation models that replicate adult dentition, typically made of rigid plastic with screw‐in teeth and elastic gingiva [1, 7, 8, 9]. While this instructor‐led approach provides direct feedback, it does not actively involve students in evaluating their own work, limiting opportunities to develop self‐assessment skills and reflective practice. Modern pedagogical methods emphasise active and collaborative learning, shifting greater responsibility to students for their own progress [10]. These methods are associated with improved critical thinking, teamwork, and student satisfaction, which support better learning outcomes and academic success—key components of clinical proficiency [11, 12].

A key skill for dental students is the ability to assess and correct their own errors, ensuring high‐quality care [2, 3, 6, 13, 14, 15]. Self‐assessment involves judging one's work against predefined standards, as described by Boud & Falchikov [16]. Andrade & Boulay further characterise self‐assessment as a formative assessment process in which students reflect on the quality of their work and evaluate how well it aligns with explicitly stated goals or criteria [17]. It is a formative process where students reflect on their work and evaluate how well it meets specific goals [5, 6, 18]. Accrediting bodies like the commission on dental accreditation emphasise the need to integrate self‐assessment into early dental education to develop these essential skills [2, 3].

One limitation of traditional simulators is that students typically start with an intact tooth for preparation, meaning they can only self‐assess their own work without exposure to a broader range of preparation errors or conditions [7]. As a result, students are limited to identifying their personal mistakes rather than developing a comprehensive understanding of common preparation defects. This lack of standardisation reduces opportunities for building a mental library of errors, which is essential for mastering self‐assessment and correction skills [7, 9, 19, 20]. 3D printing technology offers a promising alternative by creating realistic, consistent models that allow students to practice on standardised defects, enhancing both self‐assessment and error correction skills [8, 21, 22, 23, 24]. Since self‐assessment requires knowing the true quality of the evaluated work, placing students in a simulated self‐assessment scenario using standardised defective preparations ensures consistent and controlled conditions for developing evaluative skills [4, 7].

This study aims to evaluate the self‐assessment and defect correction abilities of fourth‐year dental students in fixed prosthodontics, leveraging 3D printing technology and structured rubric‐based tools. The primary objective is to assess the students' accuracy in self‐assessing their prosthetic preparations. Two secondary objectives are addressed: first, to measure their ability to correct objectified defects on teeth preparations, and second, to evaluate their satisfaction with these innovative pedagogical exercises.

## 2 Materials and Methods

### 2.1 Ethics Approval and Study Population

This study was approved by the Research Ethics Committee of the Federal University of Toulouse, ensuring compliance with ethical protocols for research involving humans (Project 2022_485). The participants were fourth‐year students from the Odontology Department at the Faculty of Health, XXXXXXXX, during the 2022–2023 academic year. Participation was voluntary, with all students providing written informed consent.

A priori power analysis was conducted using G*Power (version 3.1.9.7) to determine the required sample size for paired comparisons of two dependent means. The analysis assumed a medium effect size (*d* = 0.3), a significance level (α) of 0.05 (two‐tailed), and a desired statistical power (1‐β) of 0.80. Results indicated that a minimum of 90 participants was needed to achieve adequate power while maintaining a 5% Type I error rate.

### 2.2 Simulation Models and 3D Printing

A junior practitioner (AT) prepared eight teeth on simulators (ANA‐4 Type Typodont models maxillary and mandibular, Frasaco GmbH, Tettnang, Germany) with standardised defects to simulate common crown preparation errors. The teeth included maxillary (14, 16, 24, 26) and mandibular (36, 34, 44, 46) arches, identified using the FDI notation system.

**FIGURE 1:**
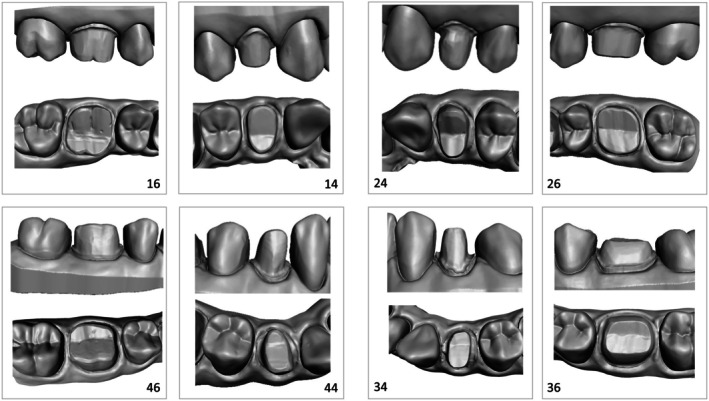
3D vestibular and occlusal view of the eight prepared teeth with defaults (16, 14, 24, 26, 36, 34, 44, 46: FDI notation).

The standardised defects included insufficient reduction, suboptimal taper, poor finishing, misaligned cusp positioning, improper cervical margin placement, and irregularities. These defects were designed to replicate common challenges encountered in crown preparations (Figure 1).

The prepared models were digitised using a desktop scanner (E3 scanner, 3shape, Copenhagen, Denmark) with a precision of 7 μm, in standard tessellation language (STL) files. The maxillary and mandibular arches were then 3D‐printed using a liquid crystal display (LCD) printer (Photon Mono, Anycubic, Shenzhen, China) with a grey coloured resin (water washable photopolymer resin, Elegoo, Shenzhen, China). The printer operated with a printing speed of 50 mm/h, and resolutions of 0.05 mm in the XY plane and 0.01 mm in the Z‐axis.

This process ensured that all students received identical 3D‐printed models with consistent defects, providing a standardised foundation for the evaluation and correction exercises.

### 2.3 Eight Preparation Defaults Evaluation

The eight prepared teeth were scored independently by two professors of prosthetics (CB and AG) and a junior practitioner (AT) to establish a reliable and consistent reference score. The evaluation was conducted using the preparation evaluation table (PET), an analytical self‐assessment grid derived from the *Rubric System* developed by Habib [25]. The PET evaluates seven criteria per preparation: occlusal reduction, axial reduction, tapering, margin placement, two‐plane reduction, finish and preservation of adjacent teeth.

Each criterion was scored on a scale of 1–4 or 1–3, depending on its complexity. The total score for all eight preparations was aggregated, resulting in a maximum possible score of 200.

To ensure consistency, the three evaluators standardised their scoring methodology before conducting independent evaluations of the preparations. Discrepancies in scoring were discussed collectively, and a consensus was reached to establish a common faculty score, which served as the gold standard for each criterion (Table A1). This faculty score was used as the reference to evaluate the students' performance.

### 2.4 Practical Session Organisation

**FIGURE 2:**
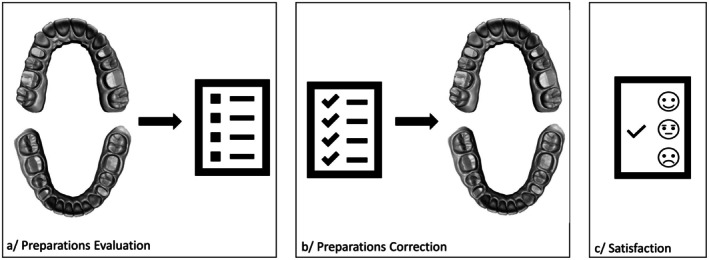
Practical work (PW) in 3 parts. (a) Evaluation of 8 crown preparations on 2 3D‐printed dental arches. The grades were filled in by the students on the Preparation Evaluation Table (PET). (b) Based on the corrected PET, the defects of the crown preparations were corrected. (c) A satisfaction questionnaire filled in about the hypothesis testing PW intended to develop evaluation skills.

The 1.5‐h session was divided into three parts (Figure 2). Students were instructed to consider the prepared teeth as if they were their own, simulating a self‐assessment scenario aimed at developing evaluative judgement in a standardised context.

### 2.5 Evaluation (40 min)

Students analysed the eight prepared teeth on the 3D models and scored each criterion using the PET (Table 1). To support their evaluation, they were provided with the PET and an analytical self‐assessment grid (Table 2). The evaluation was conducted using a clinical set comprising a dental probe and a mirror, allowing for a precise inspection of the prepared surfaces. Additionally, calibrated diamond burs were available.

**TABLE 1:** Preparation Evaluation Table (PET) for the 8 crown preparations given to the students at the beginning of the practical work (FDI World Dental Federation notation).

Parameters evaluated	Teeth							
	T16	T14	T24	T26	T34	T36	T44	T46
Occlusal reduction								
Axial reduction								
Taper								
Margin placement								
Two‐plane reduction								
Finish, margins and walls								
Preservation of adjacent teeth								

**TABLE 2:** Self‐assessment analytical grid derived from the rubric system of Habib for all‐ceramic crown preparation [25].

Parameter	Grades			
	4 points	3 points	2 points	1 point
Occlusal reduction	Supporting cuspid (2 mm) guiding cuspid (1.5 mm)	Moderately underprepared (< 1.5 mm)	Moderately over‐prepared (> 2 mm)	Severely over or under prepared (> 3 mm or < 1 mm)
Axial reduction	Optimal reduction (1.5 mm)	Moderately under‐reduced (< 1.5 mm)	Moderately over‐reduced (> 2 mm)	Severely over‐ or under‐reduced (> 2.5 mm or < 1 mm)
Taper	Optimal taper retentive walls of 6°. of taper	Near parallel walls or – over‐tapered on mesial or distal (> 8° < 16°)	Undercuts visually present or Over‐tapered on buccal or lingual (> 8° < 16°)	Severe undercuts present or Severe over‐tapered on any axial surface (> 16°)
Margin placement	Optimal position (juxta or 0.5 mm supragingival)	Moderately extended (0.5 mm intrasulcular) or under‐extended (< 1 mm supragingival)	Significantly extended (< 1 mm intrasulcular) or under‐extended (< 1.5 mm supragingival)	Severely extended (> 1 mm intrasulcular) or under‐extended (> 1.5 mm supragingival)
Two‐plane reduction		Correct angulation (placement of a prosthetic part of sufficient thickness)	Moderately incorrect angulation (under‐ or over‐prepared)	Significantly incorrect angulation (under‐ or over‐prepared)
Finish, margins and walls		Optimal finish Margins and walls are smooth Margins are continuous, well define	Moderate roughness Moderate roughness of margins and walls Margins are moderately non‐continuous, moderate lack of definition	Significant roughness Significant roughness of margins and walls Margins are non‐continuous Lack of definition of finish line
Preservation of adjacent teeth		Adjacent teeth are unaffected	Adjacent teeth are minimally touched	Adjacent teeth are abraded and flattened

### 2.6 Correction (40 min)

Students corrected the identified defects on the same 3D models using rotary instruments such as high‐speed handpieces (turbines) and low‐speed contra‐angles, equipped with diamond burs. The corrected PET was distributed after the evaluation phase to ensure all students started the correction phase with a standardised reference.

### 2.7 Satisfaction Questionnaire (10 min)

Students completed a 12‐item questionnaire anonymously to evaluate the relevance and utility of the exercise. The questionnaire assessed the usefulness of the self‐assessment, the benefits of using 3D models, and the perceived value of the correction tasks.

### 2.8 Faculty Evaluation

#### 2.8.1 Self‐Assessment Evaluation

The difference between the students' self‐assessment scores and the faculty scores was calculated as the Student‐Faculty gap (S‐F gap) [15].

- A negative S‐F gap indicates that the student underestimated the quality of their preparation compared to the faculty.
- A positive S‐F gap indicates that the student overestimated the quality.
- An S‐F gap of 0 signifies no difference between the student and faculty evaluations.

To account for cases where negative and positive gap scores cancelled each other out, absolute gap scores were calculated, reflecting the magnitude of the discrepancy regardless of its direction.

#### 2.8.2 Correction Ability

For the first secondary objective, students corrected the defects identified on the 3D models after receiving the corrected PET. The corrected models were graded by the faculty:

- 0 for a criterion poorly corrected or not corrected.
- 1 for an adequately corrected criterion (fulfilling the indications of the first column of the self‐evaluation analytical grid).

The total correction score per tooth was calculated, and an overall score of 56 (eight preparations, seven criteria per preparation) was established for the entire 3D model.

#### 2.8.3 Evaluation of Students' Satisfaction

For the second secondary objective, a satisfaction questionnaire was completed anonymously by the students during the final 10 min of the session. The questionnaire, adapted from a previous study comparing students' perceptions of 3D‐printed and stock models in paediatric dentistry, consisted of 12 statements (Table A2) [22].

The statements focused on:

- The usefulness of the self‐assessment exercise.
- The benefits of using 3D‐printed models.
- The overall relevance and utility of the correction phase.

Students rated each statement on a scale of 0–10, with 0 indicating strong disagreement and 10 indicating strong agreement. Under each number, students ticked a box to reflect their opinion.

### 2.9 Statistical Analysis

Data for the primary and first secondary objectives were analysed using TIBCO Data Science—Statistica, TIBCO Software Inc. Results were expressed as mean ± standard deviation and percentages. Tooth preparation assessments were scored according to the criteria of the PET (Table 1), which included seven specific parameters such as occlusal reduction, axial reduction, taper, margin placement, two‐plane reduction, finish, and preservation of adjacent teeth. Scores for each criterion were summed to obtain a total score per tooth, and the cumulative scores for all eight teeth were analysed using a two‐way repeated measures analysis of variance (ANOVA). The ANOVA factors included two groups (students and faculty) and eight teeth (16, 14, 24, 26, 36, 34, 44, 46, as per FDI notation).

Further, the effect of correction was evaluated with an additional two‐way repeated measures ANOVA with two factors: time point (two levels: before and after the correction) and tooth (Eight levels: 16, 14, 24, 26, 36, 34, 44, 46). For this analysis, the self‐assessment scores were transformed into binary values to ensure consistency with the correction evaluation. Criteria evaluated were scored as 1 for a score of 3 or 4 and 0 for a score of 1 or 2. This transformation allowed for a maximum score of 7 points per tooth, resulting in a total maximum score of 56 points for all eight teeth. In the case of significant main effects, a post hoc Tukey test was performed to identify specific differences. A significance level was set at *p* < 0.05.

## 3 Results

The entire fourth‐year dental class (87 students) participated in the study, with a gender distribution of 58 women (66%) and 29 men (33%). All students completed the self‐evaluations, achieving a 100% response rate.

Globally, the faculty rating of the 3D‐printed models resulted in an average score of 155/200 (77.50%). In comparison, the students' self‐assessment yielded an average score of 136.28/200 (68.10%), representing an average underestimation of 9.4% relative to the faculty evaluation. Amongst all participants, 80 students (92%) underestimated the preparations (negative S‐F gap), five students (5.70%) overestimated the preparations (positive S‐F gap), and only two students (2.30%) achieved an accurate self‐assessment. The absolute S‐F gap averaged 25.21 points out of a total of 155 points scored by faculty members, or 12.60%.

**FIGURE 3:**
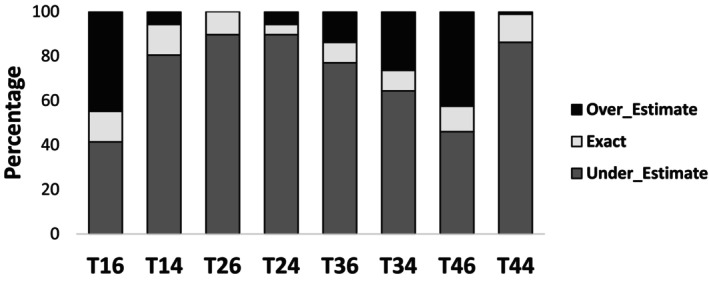
Distribution of overestimation, underestimation, and accurate self‐assessments for the eight teeth studied. The proportions are shown as percentages for each tooth.

From a tooth‐by‐tooth perspective, the proportion of students who overestimated, underestimated, or provided an exact self‐assessment is shown in Figure 3. Across all teeth, underestimation dominated, with slight variations observed between teeth. For example, tooth 44 exhibited the highest rate of underestimation, while tooth 14 showed a relatively higher percentage of exact assessments. Overestimations were consistently low across all teeth.

Regarding the evaluation criteria, the absolute S‐F gap percentages varied. The preservation of adjacent teeth (11.78%) and two‐plane reduction (13.12%) were the most accurately self‐assessed criteria. Conversely, occlusal reduction (17.89%) and axial reduction (21.88%) posed the greatest challenges for accurate self‐assessment (Table 3).

**TABLE 3:** Percentages of absolute student‐faculty gap (S‐F gap) by criteria.

	Occlusal reduction	Axial reduction	Taper	Margin placement	Two‐plane reduction	Finish, margins and walls	Preservation of adjacent teeth
Absolute S‐F gap by criterion	17.89%	21.88%	16.27%	16.88%	13.12%	16.81%	11.78%

**FIGURE 4:**
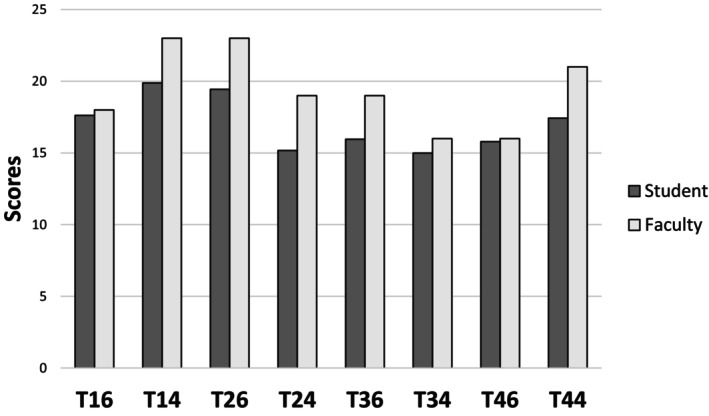
Self‐assessment scores (students) versus faculty evaluations by tooth. Significant differences were found for most teeth except T16, T34 and T46 (*p* < 0.001).

**FIGURE 5:**
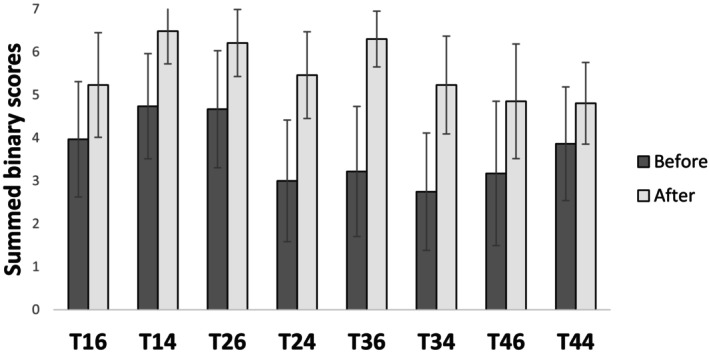
Comparison of scores before and after correction across all tooth conditions. Scores are binary for each criterion, with a maximum of 7 points per tooth. The assessment data were transformed into binary values to ensure consistency in the analysis. All corrections showed statistically significant improvements across the eight tooth conditions (*p* < 0.001).

The ANOVA analysis of the self‐assessment scores revealed significant main effects for groups (*p* < 0.001) and conditions (*p* < 0.001), with a significant interaction between groups and conditions (*p* < 0.001). Post hoc analysis indicated that students consistently scored lower than faculty, except for teeth 16, 34 and 46, where no significant differences were observed (Figure 4). After correction, a repeated measures ANOVA showed significant main effects of time (*p* < 0.001) and conditions (*p* < 0.001), along with a significant interaction between time and conditions (*p* < 0.001). Post hoc analysis demonstrated significant score improvements across all conditions after corrections (*p* < 0.001) (Figure 5).

During the correction phase, students improved the preparation scores on the 3D models. The percentages in Table 4 reflect the proportion of criteria scored at 1 (optimal) after the correction phase. While some criteria achieved high percentages of optimal scores, errors during the correction phase, such as damaging adjacent teeth, reduced the proportion of optimal scores. Finishing was the most challenging criterion, with only 58% of criteria scored as optimal, followed by occlusal reduction (74%) and margin placement (75%). The most successfully corrected criteria were preservation of adjacent teeth (96%) and axial reduction (91%).

**TABLE 4:** Percentage of criteria scored as optimal (1) after the correction phase across the seven evaluated parameters.

	Occlusal reduction	Axial reduction	Taper	Margin placement	Two‐plane reduction	Finish, margins and walls	Preservation of adjacent teeth
Percentage of optimal scores	74%	91%	89%	75%	79%	58%	96%

The satisfaction questionnaire revealed that the most positively rated statements were “Learning to detect errors should be part of practical training in fixed prosthodontics” (9.01/10), “A simulation with different clinical cases would be interesting” (8.14/10), and “The practical work carried out seems beneficial for analysing/correcting your future preparations” (8.13/10). The lowest‐rated statements were “The colour of the printout is disturbing” (2.08/10) and “The model is useful as a preparation for the clinic” (5.31/10).

## 4 Discussion

This study demonstrated that students generally underestimated their performance (92%, S‐F gap: −9.4%), with self‐assessment accuracy varying significantly across 5 of the 8 evaluated tooth types (*p* < 0.001). While students showed better accuracy in adjacent teeth preservation, greater discrepancies were observed in occlusal and axial reduction. The correction phase resulted in significant improvements across all tooth conditions (*p* < 0.001), but challenges persisted, particularly in occlusal reduction and finishing. Despite these difficulties, students expressed high satisfaction with the exercise, valuing its educational and practical relevance. However, they noted limitations in the tactile realism of 3D‐printed models.

Crown preparation requires precision, manual dexterity and three‐dimensional visualisation, which demand extensive practice. Traditional assessment methods often lack consistency, undermining student confidence and skill development (1). This study introduced standardised 3D‐printed models with predefined defects, replicating clinical scenarios to support self‐assessment and error correction. These findings align with prior research demonstrating that structured rubrics and realistic models enhance learning and readiness for clinical practice [8, 26, 27, 28, 29, 30, 31, 32, 33].

### 4.1 3D‐Printed Model

3D‐printed models facilitated objective assessments, enabling repeated practice and standardisation in preclinical scenarios [7]. They raised awareness of medical errors and promoted skill development, addressing limitations in traditional assessments [21]. However, tactile differences compared to natural teeth or Frasaco models posed challenges, highlighting areas for improvement [21, 34].

### 4.2 Challenges in Self‐Assessment

Students identified significant challenges in self‐assessment during preclinical training, particularly issues of subjectivity and inconsistency when evaluations relied on instructors [7]. Discrepancies often occurred when different instructors assessed the same task, leading to variable feedback and potential confusion. These findings underscore the need for objective evaluation tools to ensure consistency and reliability in assessments [21].

Building on prior research, this study introduced standardised protocols using 3D‐printed models of maxillary and mandibular teeth. By providing identical tasks under consistent conditions, this approach minimised comparison bias—a common issue in self‐assessment studies—and enabled fairer comparisons and more reliable insights into student accuracy [35].

Although referred to as self‐assessment, the exercise did not involve students evaluating their own work. Instead, it simulated a self‐assessment scenario by assigning students the task of evaluating standardised defective preparations. True self‐assessment typically requires expert feedback to calibrate judgement and limits exposure to only one's own errors [7, 21, 25]. While assessing others' work may engage different cognitive processes, it remains effective for developing evaluative skills in a controlled setting [4]. This approach ensured control over defect types, reduced bias, and provided a consistent framework for training self‐assessment skills.

### 4.3 Performance and Self‐Assessment Accuracy

Students predominantly underestimated the quality of their preparations, with 92% showing a negative S‐F gap compared to faculty evaluations. This contrasts with other studies in dental education, where students often overestimate their abilities [3, 6, 15, 35, 36]. The discrepancy observed here likely stems from the specific focus on defect detection, directing students' attention to flaws rather than overall quality. Limited clinical experience further contributed to this trend.

Although cognitive biases, such as the tendency of lower‐performing students to overestimate their abilities and higher‐performing students to underestimate theirs, have been observed in healthcare education [2, 6, 13, 15], this was not specifically analysed in our study. Similarly, prior research has highlighted gender differences in self‐assessment accuracy, where females tend to underestimate their performance and males often overestimate theirs, potentially influenced by societal or cultural factors [6, 37, 38, 39]. Future research should explore these aspects to better understand their influence on self‐assessment in dental education.

### 4.4 Implications for Preclinical Education

The observed absolute S‐F gap of 12.6% underscores the need for improved self‐assessment training in preclinical education, particularly for challenging criteria like occlusal and axial reduction. Incorporating structured self‐assessment exercises supported by clear rubrics can foster autonomy, reflective practices, and consistency amongst evaluators [5, 11, 36]. Such approaches might enable students to align their evaluations more closely with faculty standards and build confidence in their skills.

Longitudinal studies could investigate how structured self‐assessment practices influence skill development over time, particularly in diverse student populations. Additionally, future research should expand to include cognitive biases and gender differences in self‐assessment, as these factors could inform tailored training interventions. Emerging technologies, such as virtual reality, haptics and AI, also hold promise for enhancing self‐assessment practices and optimising dental education.

### 4.5 Evaluation of Correction Capacity

Identifying and correcting preparation defects is essential for developing clinical competence in fixed prosthodontics. Students faced challenges in occlusal reduction and finishing, often due to a limited understanding of their biomechanical relevance [40]. While 3D‐printed models provided consistency, students frequently focused on individual teeth rather than their relationships within the dental arch. Tools like reduction silicone indices and antagonist occlusion analysis could enhance understanding and bridge the gap between preclinical and clinical practice [11, 41].

The correction exercise aligned with clinical scenarios by requiring students to evaluate and improve preparations before impressions. This iterative process between evaluation and correction, supported by structured self‐assessment and targeted feedback, improved technical performance and prepared students to meet clinical expectations.

### 4.6 Student Satisfaction

Students expressed high satisfaction with 3D‐printed models, highlighting their benefits for fostering learning and preparing for clinical practice. Consistent with Marty et al. [22], students preferred 3D‐printed models derived from real cases over traditional standard models, as seen in paediatric dentistry. In this study, students emphasised the value of practical exercises for error detection and correction, considering them highly relevant to their future clinical practice. The highest satisfaction scores were recorded for statements like, “Learning to detect errors should be part of practical training in fixed prosthodontics” and “A simulation with different clinical cases would be interesting.” These results underscore the importance of integrating error detection into preclinical training to bridge the gap between theoretical exercises and real‐world application [36].

### 4.7 Strength and Limitation

This study combines objective self‐assessment evaluations with student experiences, emphasising the importance of reproducible cases to build a “library” of defects for practice and correction. This competency‐based approach not only enhances clinical competence but also fosters lifelong learning by enabling students to refine their evaluation and correction skills in controlled, standardised conditions [21, 42, 43].

However, the study's focus on a single institution and the absence of measures such as inter‐rater reliability or intra‐class correlation coefficients limit the generalizability of the findings. Additionally, relying on visual assessments without reduction keys may have impacted the accuracy of occlusal reduction evaluations. Future research should expand to include incisors, canines, and emerging technologies such as virtual reality, haptics and AI to validate these findings and further optimise dental education [21, 34].

## 5 Conclusion

This study highlights the value of incorporating structured self‐assessment and error correction exercises into preclinical dental education. Using standardised 3D‐printed models with deliberate defects, students were able to evaluate and correct crown preparation errors within a consistent and objective framework. While self‐assessment revealed a tendency to underestimate performance—particularly for challenging criteria such as occlusal and axial reduction—the correction phase demonstrated significant improvements across all teeth, despite persistent difficulties with finishing and occlusal reduction. Student feedback through the satisfaction questionnaire underscored the educational value and clinical relevance of these exercises, with the 3D‐printed models being especially appreciated for fostering critical thinking and reflective practice. These findings support the integration of standardised self‐assessment and correction exercises early in the curriculum to enhance technical skills and autonomy, while future studies should explore their broader applicability and the potential of emerging technologies to further optimise dental education.

## Conflicts of Interest

The authors declare no conflicts of interest.

## Data Availability

Data available on request from the authors.

## Appendix

**TABLE A1:** Correction of the preparation evaluation table (PET).

Parameters evaluated	Teeth							
	16	14	24	26	34	36	44	46
Occlusal reduction	1	4	1	4	1	2	3	1
Axial reduction	3	4	3	3	3	4	4	1
Taper	4	4	2	4	3	2	2	4
Margin placement	3	3	4	4	3	2	4	3
Two‐plane reduction	2	2	3	2	2	3	2	2
Finish, margins and walls	2	3	3	3	1	3	3	2
Preservation of adjacent teeth	3	3	3	3	3	3	3	3

**TABLE A2:** Satisfaction questionnaire – 3D error detection/correction models.

**A: Detecting and correcting preparation defects is complicated**										
0	1	2	3	4	5	6	7	8	9	10
□	□	□	□	□	□	□	□	□	□	□
**B: The texture is disturbing**										
0	1	2	3	4	5	6	7	8	9	10
□	□	□	□	□	□	□	□	□	□	□
**C: The colour of the print is disturbing**										
0	1	2	3	4	5	6	7	8	9	10
□	□	□	□	□	□	□	□	□	□	□
**D: The simulation model is realistic**										
0	1	2	3	4	5	6	7	8	9	10
□	□	□	□	□	□	□	□	□	□	□
**E: Detecting and correcting preparation errors on the platelet is useful to you**										
0	1	2	3	4	5	6	7	8	9	10
□	□	□	□	□	□	□	□	□	□	□
**F: A simulation with different clinical cases would be interesting**										
0	1	2	3	4	5	6	7	8	9	10
□	□	□	□	□	□	□	□	□	□	□
**G: Level of difficulty**										
0	1	2	3	4	5	6	7	8	9	10
□	□	□	□	□	□	□	□	□	□	□
**J: The model is useful as a preparation for the clinic**										
0	1	2	3	4	5	6	7	8	9	10
□	□	□	□	□	□	□	□	□	□	□
**K: I would like to have this booklet available so that I can train independently**										
0	1	2	3	4	5	6	7	8	9	10
□	□	□	□	□	□	□	□	□	□	□
**L: I would have liked to have this booklet in 2** ^**e**^ **and 3** ^**e**^ **year**										
0	1	2	3	4	5	6	7	8	9	10
□	□	□	□	□	□	□	□	□	□	□
**M: Learning to detect errors should be part of practical training in fixed prosthodontics**										
0	1	2	3	4	5	6	7	8	9	10
□	□	□	□	□	□	□	□	□	□	□

*Note:* 0: disagree; 10: strongly agree.
